# Challenges in the analytical determination of ultra-short-chain perfluoroalkyl acids and implications for environmental and human health

**DOI:** 10.1007/s00216-020-02692-8

**Published:** 2020-05-12

**Authors:** Maria K. Björnsdotter, Leo W. Y. Yeung, Anna Kärrman, Ingrid Ericson Jogsten

**Affiliations:** Man-Technology-Environment Research Centre (MTM), Örebro University, 701 82 Örebro, Sweden

**Keywords:** Ultra-short-chain perfluoroalkyl acid, Trifluoroacetic acid, Perfluoropropanoic acid, Trifluoromethane sulfonic acid, Perfluoroethane sulfonic acid, Perfluoropropane sulfonic acid

## Abstract

Ultra-short-chain perfluoroalkyl acids have recently gained attention due to increasing environmental concentrations being observed. The most well-known ultra-short-chain perfluoroalkyl acid is trifluoroacetic acid (TFA) which has been studied since the 1990s. Potential sources and the fate of ultra-short-chain perfluoroalkyl acids other than TFA are not well studied and data reporting their environmental occurrence is scarce. The analytical determination of ultra-short-chain perfluoroalkyl acids is challenging due to their high polarity resulting in low retention using reversed-phase liquid chromatography. Furthermore, recent studies have reported varying extraction recoveries in water samples depending on the water matrix and different methods have been suggested to increase the extraction recovery. The present review gives an overview of the currently used analytical methods and summarizes the findings regarding potential analytical challenges. In addition, the current state of knowledge regarding TFA and other ultra-short-chain perfluoroalkyl acids, namely perfluoropropanoic acid, trifluoromethane sulfonic acid, perfluoroethane sulfonic acid, and perfluoropropane sulfonic acid‚ are reviewed. Both known and potential sources as well as environmental concentrations are summarized and discussed together with their fate and the environmental and human implications.

## Introduction

Ultra-short-chain perfluoroalkyl acids (PFAAs) include perfluoroalkyl carboxylic acids (PFCAs) and perfluoroalkyl sulfonic acids (PFSAs) with a chain length of 1–3 fluorinated carbons. These include TFA, perfluoropropanoic acid (PFPrA), trifluoromethane sulfonic acid (TFMS), perfluoroethane sulfonic acid (PFEtS), and perfluoropropane sulfonic acid (PFPrS). Their chemical structure, chemical formula, CAS number, molecular weight, water solubility, vapor pressure, pK_a_, and logP (octanol/water) are provided in Table 1. These acids belong to the group of per- and polyfluoroalkyl substances (PFASs), a group of highly fluorinated chemicals widely used in industrial and commercial applications [1]. In the PFAS group, PFAAs commonly refers to acids with a fully fluorinated carbon chain, for example, carboxylic and sulfonic acids [2]. The use of PFASs has resulted in global environmental contamination [3] and concern has been raised because of the persistence and potential for bioaccumulation of these substances. The mutual properties of ultra-short-chain PFAAs are their low molecular weight, high polarity, and persistence to degradation. The low molecular weight and high polarity of ultra-short-chain PFAAs give them unique properties among the chemicals commonly referred to as PFASs. The polarity is a result of both a short perfluorinated carbon backbone and an acidic functional group with pK_a_ values below 1.4 suggesting that these compounds are charged at environmental pH. Due to the high polarity and water solubility of these substances, the potential for bioaccumulation is low, as has been shown for the short-chain perfluorobutane sulfonic acid (PFBS) [4]. However, both PFBS and perfluorobutanoic acid (PFBA) have been detected in humans [5, 6]. The high persistence of ultra-short-chain PFAAs will result in environmental accumulation, especially in aquatic environments, leading to potential risks for aquatic organisms and increased human external exposure through drinking water as described by Cousins et al. [7].

**Table 1:**
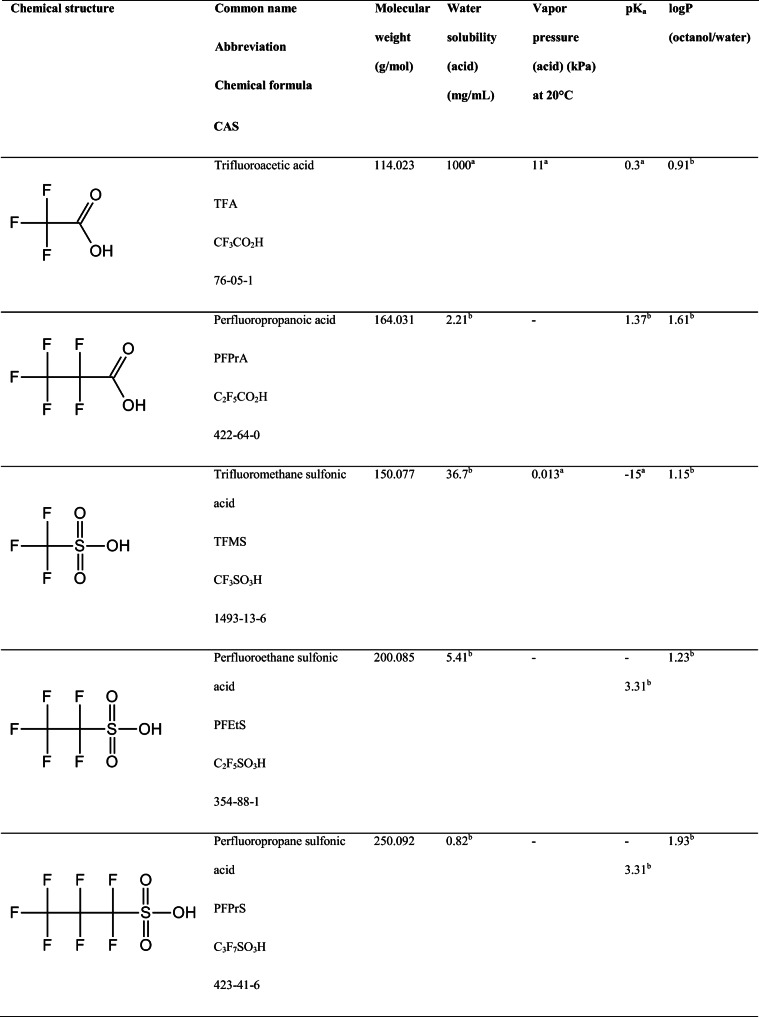
Chemical structure, name, and selected properties of ultra-short-chain PFAAs

^a^Data obtained from Pubchem (available at https://pubchem.ncbi.nlm.nih.gov/)

^b^Data obtained from Chemicalize (available at https://chemicalize.com/#/)

Increased attention was paid on TFA after the phaseout of chlorofluorocarbons (CFCs), according to the Montreal Protocol on substances that deplete the ozone layer in 1989 [8], and the subsequent introduction of hydrofluorocarbons (HFCs) and hydrochlorofluorocarbons (HCFCs). TFA has since then been widely studied and reported in various environmental matrices globally [9–27]. Other ultra-short-chain PFAAs have not been well studied and data reporting their sources, fate, and environmental occurrence is scarce. Limited available data might be partly caused by the analytical challenges to measure these substances, as they are not well retained on reversed-phase liquid chromatography columns resulting in poor separation [20]. A limited number of analytical methodologies have been developed for the analytical determination of TFA as well as other ultra-short-chain PFAAs. Extraction of PFASs including ultra-short-chain PFAAs from water is commonly done using a method based on mixed-mode hydrophobic and weak anion-exchange solid-phase extraction (WAX-SPE) [20]. However, recent studies report varying and matrix depending extraction recoveries for TFA [11, 28], and work has focused on increasing the recovery of ultra-short-chain PFAAs in complex sample matrices [29, 30]. Several reviews on PFAS summarizing the current state of knowledge and the existing analytical techniques exist; however, there are no published reviews addressing neither the challenge in the analytical determination of ultra-short-chain PFAAs nor their sources, environmental fate, occurrence, and implications. The present review assesses and summarizes existing analytical methods and the potential analytical challenges for ultra-short-chain PFAAs. In the context of measurements, the environmental occurrence, their sources and fate, and the environmental and human implications are also discussed.

### Analytical techniques and challenges

Published data on ultra-short-chain PFAAs is obtained using a variety of different analytical methods (sample preparation and instrumental analysis) that are summarized in Table 2. Analytical determination of TFA in water samples has commonly been done by gas chromatography (GC) coupled to electron capture detection (ECD) [24, 27, 36] or mass spectrometry (MS) [9, 12, 13, 22, 31, 37]. The method described by Scott and Alaee [31] for analytical determination of TFA in aqueous samples involves evaporation of samples with volumes of 500 to 1000 mL down to 50 mL by rotary evaporation followed by derivatization with 2,4-difluoroanilide. The recovery of the method based on extraction of water spiked with analytical standards was 89 ± 7%; this recovery did not take into consideration possible matrix differences that might affect the derivatization and extraction efficiency. The method has later been slightly modified and a surrogate standard (trichloroacetic acid) has been used as an internal standard for TFA [15, 18]. The recovery of TFA based on trichloroacetic acid was 95 ± 7% [15]. The limit of detection based on the average TFA concentration in blank samples was in the range 0.5 to 1 ng/L [15, 18, 19, 32]. The same method has later also been used for analytical determination of PFPrA and short-chain (C4–C6) PFCAs in addition to TFA [32].

**Table 2:** Summary of analytical methods and method performance for quantification of ultra-short-chain PFAAs in water samples

Sample preparation	Separation-detection technique	Quantification	Sample matrix	Sample volume (mL)	Instrument run time (min)	Analytes	Extraction efficiency (%) based on matrix spike	LOD/LOQ (ng/L)	Reference (publication year)
Sample volume reduction to 50 mL by rotary evaporation at 55 °C. Derivatization and salting-out LLE with ethyl acetate. Removal of water and evaporation to dryness at 30 °C. Re-dissolution in benzene/toluene followed by volume reduction to 2 mL.	GC-MSD (SIM)	Internal standard calibration using ^13^C-labeled trichloroacetic acid	Rain (*n* = 2)	500–1000	40	TFA	89 ± 7	0.1–1^a^/–	Scott and Alaee [31] (1998)^e^, Scott et al. [16] (2000)^e^, Scott et al. [19] (2002)^e^, Scott et al. [15] (2005), Scott et al. [18] (2005), Scott et al. [32] (2006)^e^, Scott et al. [17] (2006)
			Snow (*n* = 3)			PFPrA			
			Groundwater (*n* = 2)						
			Surface water (*n* = 4)						
			Subsurface water (*n* = 3)						
			Drinking water (*n* = 3)						
Extraction by anion-exchange SR Empore disk, derivatization. Surface water samples with salinity > 500 μS were cleaned up by LLE prior to extraction.	GC-ECD	External standard calibration	Rain (*n* = 2)	400	30	TFA	Rain 97 ± 4	32/36	Wujcik et al. [33] (1998)
			Tap water (*n* = 2)				Tap water 105 ± 0.3		
			Surface water (*n* = 6)				Surface water 102 ± 4		
WAX-SPE	Ion-exchange HPLC-MS/MS	Internal standard calibration using ^13^C-PFBA and ^13^C-PFOS	Rain (*n* = 4)	100	30	TFA	76 ± 9	–/0.5^b^	Taniyasu et al. [20] (2008)
						PFPrA	105 ± 1	–/0.1^b^	
						PFEtS	105 ± 3	–/0.1^b^	
						PFPrS	105 ± 4	–/0.5^b^	
WAX-SPE	SFC-MS/MS	–	Rain (*n* = 2)	200	8	TFA	79 ± 10	–/0.2–0.5^b^	Yeung et al. [34] (2017)
			Surface water (*n* = 2)			PFPrA	84 ± 8		
						PFEtS	93 ± 7		
						PFPrS	85 ± 4		
WAX-SPE after adjusting the pH to 3.8–4.0	RP-LC-ESI-MS/MS	Internal standard calibration using ^13^C-TFA	Spring water (*n* = 1)	50	20	TFA	Tap water 97 ± 0.7	5.5/26^c^	Janda et al. [11] (2018)
						PFPrA		0.9/4.3^c^	
			Tap water (*n* = 21)				Groundwater 99 ± 4		
			Groundwater (*n* = 42)				Surface water 101 ± 1		
			Surface water (*n* = 43)						
							Tap water 95 ± 2		
							Groundwater 83 ± 2		
							Surface water 104 ± 5		
Direct injection	SFC-MS/MS	Internal standard calibration using ^13^C-PFBA	Surface water	0.25	11	TFA	81 ± 0.4	34^d^/–	Björnsdotter et al. [35] (2019), Björnsdotter et al. [28] (2019)
			Groundwater						
			Landfill leachate						

“–” not reported

^a^LOD based on repeated procedural blanks

^b^Instrumental LOD

^c^LOD/LOQ according to DIN 32645

^d^LOD based on repeated instrumental blanks

^e^Did not include the use of ^13^C-labeled trichloroacetic acid

A modification of existing methods for TFA extraction from aqueous samples with the aim to reduce the time needed for extraction (e.g., rotary evaporation of aqueous samples and long derivatization reactions) and eliminate the use of hazardous reagents was developed by Wujcik et al. [33]. The extraction of TFA from 200 to 400 mL water samples was done using anion-exchange SR Empore disks. The extracts were analyzed using headspace injection onto a GC-ECD system after derivatization of TFA. Test extractions with water containing NaCl and Na_2_SO_4_ at different concentrations showed a decreased recovery of TFA at higher salinity which was a result of competing anions during the anion-exchange Empore disk extraction. The recovery started to decrease at a concentration of 120 mg NaCl in 400 mL water. For this reason, an additional step based on salting-out liquid-liquid extraction (LLE) was included prior to extraction for samples with a high salinity (conductivity > 500 μS) in order to obtain good recovery of TFA. The recovery of TFA in different test samples (rain water, tap water, and surface water), some of which the additional LLE cleanup was applied on, was found to be 102 ± 4%.

Taniyasu et al. [20] developed a method based on weak anion-exchange solid-phase extraction (WAX-SPE) followed by ion-exchange high-performance liquid chromatography (HPLC) coupled to tandem mass spectrometry (MS/MS) for the analytical determination of 29 PFAAs in water samples. After extraction, the 29 PFAAs including the ultra-short-chain PFAAs TFA, PFPrA, PFEtS, and PFPrS were separated on an ion-exchange RSpak JJ50 2D column under isocratic conditions with 80% methanol at 0.3 mL/min during 30 min. The recovery of the WAX-SPE method was evaluated based on extraction of MilliQ water spiked with analytical standards and was 99 ± 5% (TFA), 96 ± 8% (PFPrA), 95 ± 6% (PFEtS), and 96 ± 7% (PFPrS). The recovery of the method when applied on rain water was 105 ± 1% (PFPrA), 105 ± 3% (PFEtS), and 105 ± 4% (PFPrS). However, the recovery of TFA was slightly lower (76 ± 9%) in rain water indicating some losses during the extraction due to the sample matrix. External calibration curve was used for quantification. The limits of quantification (LOQs) were based on the lowest concentration of the compound injected within the linear range that resulted in a reproducible measurement. The LOQ was 0.5 ng/L (TFA, PFPrS) and 0.1 ng/L (PFPrA, PFEtS). The same study also investigated the separation of the 29 PFAAs on two reversed-phase (RP) C18 columns. Poor retention and poor separation of C2–C4 PFAAs was observed and TFA was not retained on one of the columns showing that RP-LC is not a suitable technique for the separation of ultra-short-chain PFAAs [20].

In contradiction to the conclusion by Taniyasu et al. [20] that RP-LC is not a suitable technique for the analytical determination of ultra-short-chain PFAAs, Janda et al. [11] developed a method for the determination of C2–C8 PFCAs by WAX-SPE followed by RP-LC-MS/MS. The chromatographic separation of C2–C8 PFCAs was assessed using two different LC columns, one mixed-mode column offering both ion-exchange and hydrophilic interactions (Obelisc N) and a core-shell RP column (Kinetex C18) [11]. The separation on the Obelisc N column was influenced by co-extracted interferences in environmental samples while successful separation was achieved with the Kinetex C18 column using formic acid as an additive in the mobile phase to increase the retention of TFA and PFPrA. Signal suppression due to co-extracted matrix components using electrospray ionization was observed for all PFCAs (C2–C8) using electrospray ionization. The variation of the signal suppression for C3-C8 PFCAs was below 9% while the variation for TFA was 41%. This highlights the need of measures for reliable quantification of TFA, for example, isotope dilution with labeled internal standard or standard addition.

A method using supercritical fluid chromatography (SFC) coupled to MS/MS was developed by Yeung et al. [34] for the analytical determination of several PFASs including TFA, PFPrA, PFEtS, and PFPrS in rain and river water samples. Eight different SFC columns were investigated of which six resulted in satisfactory separation of C2–C14 PFAS. The best result was obtained using a Torus diol column, which showed better separation and resolution. The developed SFC-MS/MS method was applied on rain and surface water samples after extraction by WAX-SPE using the method described by Taniyasu et al. [20]. The recovery of the ultra-short-chain PFAAs based on spiked rain water was 79 ± 10% (TFA), 84 ± 8% (PFPrA), 93 ± 7% (PFEtS) and 85 ± 4 (PFPrS). The repeatability and reproducibility of the method at 1 ng spike level ranged from 84 to 98% and 86–98%, respectively. The LOQ was defined as the lowest spiked level of PFAS with a signal-to-noise ratio of ten and ranged from 0.2 to 0.5 ng/L. External calibration curve was used for quantification. The method by Yeung et al. [34] has later been modified and does now also include TFMS [35].

Analytical techniques developed for TFA and other ultra-short-chain PFAAs have different strengths and limitations. GC separation offers increased performance compared with LC but the analytes in question need derivatization, which increases the time needed for sample preparation as well as adds one chemical reaction that can affect the method recoveries and robustness. Furthermore, using MS/MS over MS increases the selectivity and often the sensitivity since measuring product ions decreases the chemical noise. Analytical determination by SFC is the most time-efficient separation technique reported as the analysis time is only 8–11 min. SFC also consumes relatively little organic solvent as the mobile phase mainly consists of supercritical carbon dioxide with organic solvent added as modifier. One limitation could be that SFC instrumentation is not available as frequently as LC instrumentation in commercial and research laboratories and investment of a new chromatographic system would be at a considerable cost. Many laboratories aim to determine a number of different PFAS classes. For this reason, methods that enable several PFAS classes to be analytically determined simultaneously are beneficial. Good separation of C2–C14 PFCAs and C2–C10 PFSAs using SFC has been shown [34]; however, separation of structural isomers was not demonstrated. Separation of structural isomers may be relevant for short- and long-chain PFASs and can be achieved using RP-LC [38]. Consequently, methods that allow for separation of ultra-short-chain, short-chain and long-chain PFASs, including structural isomers, are favorable. RP-LC may be a suitable candidate as the technique has been successfully applied for separation of C2–C8 PFCAs [11] and separation of structural isomers of PFOS and perfluorooctanoic acid (PFOA) [38]. Successful separation of C2–C8 PFCAs using RP-LC was demonstrated by Janda et al. [11], although inadequate retention of C2–C3 PFAAs has been reported [20]. The reproducibility of the RP-LC method for C2–C8 PFASs was not evaluated in terms of analytical instrumentation and consumables (e.g., chromatographic column) [11]. The chromatographic retention of C2 and C3 PFCAs was limited and might vary among columns and depend on sample matrix.

Different extraction techniques include ion-exchange-based methods or LLE. The former allow large-volume samples to be extracted and concentrated at the same time while the latter often requires a concentration step by rotary evaporation. Furthermore, extraction of aqueous samples by WAX-SPE allows for simultaneous extraction of a wide range of PFASs including long-, short-, and ultra-short-chain PFAAs as well as neutral PFASs [34] and many samples (10–15) can be processed at the same time [11, 20]. The extraction time with WAX-SPE depends on volume of the sample and the sample matrix; filtration of water sample prior to extraction is needed when samples containing large amounts of particles and/or biological material may result in clogging the SPE cartridges. Concentration by rotary evaporation is a tedious process and a limiting factor for sample throughput as a single sample of 250 mL takes about 45–120 min to evaporate [33]. Direct injection may be a suitable technique for analytical determination of TFA and PFPrA in environmental samples such as rain and surface water, as the concentration observed in these waters may be expected to be sufficiently high that concentration step is not needed. Analytical determination by direct injection offers a high sample throughput as very little sample preparation is required [28]. However, analytical determination of TFA in drinking water samples requires sample concentration, as the expected concentration is low. The direct injection method was only tested on TFA and quantitative determination of other ultra-short-chain PFAAs may require sample concentration as the environmental concentration might not be sufficiently high for direct injection.

The extraction efficiency for both TFA and PFPrA by WAX-SPE has been found to be affected by the pH of the samples. Janda et al. [11] evaluated two different weak anion-exchange mixed-mode SPE materials for the extraction of PFCAs from water, namely Strata X-AW and Oasis WAX. The effect of pH was found to be more prominent for TFA compared with PFPrA. The Oasis WAX sorbent was considered more robust as acceptable recoveries (95–103%) of TFA was observed over a broader pH range (pH 3–5) compared with Strata X-AW (pH 3). Acceptable recoveries (93–114%) of PFPrA were observed at pH 3–5 with both sorbents but decreased to 36% at pH 6 with Strata X-AW. The authors suggested that the influence of pH on the extraction recovery can be explained by the competition of anions (such as bicarbonate which becomes carbonic acid at pH 4.3) present in the sample matrix [11]. Varying extraction efficiencies of TFA in water samples by WAX-SPE was also reported by Björnsdotter et al. [28]. It was also suggested that the extraction efficiency of TFA by WAX-SPE does not only depend on the pH but rather a combination of the pH and sample matrix. To overcome this, a method based on direct injection analysis with SFC-MS/MS for the determination of TFA in water was suggested in order to avoid losses during extraction [28], as was illustrated by comparing direct injection with WAX-SPE. About 20% signal suppression during electrospray ionization was observed for TFA using the direct injection method and was constant for the different matrices tested (landfill leachate, surface water, and groundwater). This emphasizes the need for authentic labeled standards or standard addition for the quantification of TFA in different water matrices.

Two recent studies reported low recovery of ultra-short-chain PFAAs in samples after chemical oxidation at high pH using persulfate and sodium hydroxide (i.e., total oxidizable precursor (TOP) assay) [29, 30]. The TOP assay results in a solution containing large amounts of SO_4_^2−^ and the pH is around 10; before SPE, the pH has to be adjusted to 4 using HCl. The oxidation and presence of ions complicate the quantitative determination of ultra-short-chain PFAAs both by reducing the extraction recovery in anion-exchange based extraction methods and/or by lowering the MS ionization efficiency. The low extraction recovery of TFA in aqueous samples with a high salinity was also reported before [33]. Wang et al. [30] reported a method based on pretreatment with Cleanert ion chromatography (IC)-Ba/Ag/H cartridges to remove excessive SO_4_^2−^ or Cl^−^ in the solution after TOP assay. The recovery of TFA in test samples increased from 0% to 78% when the IC-Ba/Ag/H cartridges were applied prior to extraction by WAX-SPE. The results were obtained using mass-labeled PFBA as internal standard which has been shown to not have the same ionization efficiency as TFA using ESI [28].

The detection and quantification limits (LOD and LOQ) reported with the different techniques are in the same range. However, the majority of studies have reported the LOD and/or LOQ based on the linear range of the instrument even if the concentration of TFA observed in the blank was not well below the LOQ of the instrument. TFA can be expected to be ubiquitous in the atmosphere and blank concentrations can be expected to be high and varying between batches of samples. Different approaches have been carried out in order to lower the blank contamination with TFA. Some studies have used purified water prepared in the laboratory. The purified water was prepared by passing tap water through a semipermeable membrane and then through a LC-NH_4_-SPE tube [16, 31]. Repeated blanks contained 0.005 [31] and 0.025 [16] ng TFA using purified water. However, it is not clear if this method resulted in lower TFA blank contamination or not, since the TFA concentration was not reported in the tap water or in the semipermeable received water. Laboratory materials can also be a source for ultra-short-chain PFAAs. Scott et al. [19] tested the leaching of TFA from Teflon caps that were submerged in water for several weeks. No increase in TFA was observed. In contrast, both perfluoroalkoxy and polytetrafluoroethylene (PTFE, e.g., Teflon®) polymers were shown to leach TFA when submerged in water in amber bottles and stored at room temperature for 2 months [33]. Therefore, fluorinated polymers should be avoided during sampling, storage, and analytical determination of TFA (and other short-chain PFCAs). In addition to avoiding fluorinated polymers, Wujcik et al. [33] cleaned all glassware in several steps including hot tap water with and without soap, deionized water, and acetone. The glassware was then baked at 150 °C for 3 h and immediately covered with aluminum foil to prevent condensation. After cleaning and baking, repeated blanks contained 12.7 ng TFA, but it is not reported whether or not this procedure resulted in lower TFA contamination. Berg et al. [9] reported that despite precautions, contamination during sample preparation could not be completely avoided. Investigations showed that TFA among other haloacetic acids was present in ambient air and in sulfuric acid and methyl tert-butyl ether that were used in the extraction. Many studies have applied precautions during sample preparation, but few studies report contamination sources. It is possible that a large contribution of TFA blank contamination originates from the ambient air, and in that case, the contamination might depend on season [22, 23] and the diurnal cycle [23]. Other potential sources including solvents, chemicals, laboratory equipment, and consumables must be taken into consideration and careful blank tests should be done prior to analytical determination.

Environmental occurrence of TFA has been frequently reported since the mid-1990s. Published data is obtained using a variety of different analytical methods (sample preparation and instrumental analysis) and the majority of data has been obtained without using mass-labeled TFA as internal standard. Quantification of ultra-short-chain PFAAs is commonly done by using a mass-labeled standard for PFAAs with longer carbon chain, e.g., mass-labeled PFBA for quantification of TFA and PFPrA. However, the extraction and ionization efficiencies may not be similar resulting in errors which were reported for the quantification of TFA using mass-labeled PFBA as a surrogate standard [28]. Some studies used other surrogate standards (e.g., trichloroacetic acid) or external calibration curve. Different extraction efficiencies and quantification methods make comparison of data difficult. For these reasons, efforts should be made on accurate and reproducible analytical techniques. The addition of acid to the sample would reduce the competition of anions (such as bicarbonate) as well as break up ion pairs that could be formed between TFA and matrix components. The extraction efficiency should be investigated for each type of sample matrix and the use of a high purity, mass-labeled internal standard for TFA as well as other ultra-short-chain PFAAs would increase the quality and comparability of published results. Since TFA is a degradation product of volatile HFCs globally distributed and can be expected to be found in the laboratory environment, precaution should be taken to ensure that any mass-labeled standard used is not contaminated with native TFA.

### Reported environmental concentrations

Concentrations of TFA has been reported in various abiotic environmental matrices globally. Reported concentrations of TFA in precipitation and in surface water, using different analytical methods, are listed in Tables 3 and 4. TFA has been reported in precipitation at concentrations ranging from < 0.1 ng/L to 2.4 μg/L [9, 12, 16–18, 20–22, 24, 25, 27, 33]. The highest concentration of TFA (2.4 μg/L) was measured in precipitation near an urban area in the USA [17]. Yet, TFA seem to be ubiquitous in precipitation even at very remote sites [16, 22]. TFA was the most abundant PFCA in Japan [20], the USA and in Canada [17]. In the precipitation collected in the USA and in Canada, TFA was detected in all samples analyzed (*n* = 196). A 17-fold increase from 23 to 98 ng/L to 345–828 ng/L was observed for TFA concentrations in an urban landscape waters in China between 2002 and 2012 [25], indicating that the environmental concentrations of TFA are increasing, likely as a result of the introduction of HFCs after the phase out of ozone depleting chlorofluorocarbons (CFCs).

**Table 3:** Reported concentrations of TFA and PFPrA (ng/L) in precipitation using different analytical techniques

Sampling year	Country	Number of samples (n)	Concentration range (ng/L)	Analytical technique	Reference (publication year)
TFA					
1995–1996	Germany	20	10–410	Derivatization, LLE, GC-MS	Jordan and Frank [12] (1999)
1996	Poland	4	26–1100	Rotary evaporation, derivatization, GC-MS	Von Sydow et al. [22] (2000)
1996	Ireland	8	2–92	Rotary evaporation, derivatization, GC-MS	Von Sydow et al. [22] (2000)
1996–1997	The USA	60	21–760	Anion-exchange SR Empore disk, derivatization, HS-GC-ECD	Wujcik et al. [24] (1999)
1996–1997	Switzerland	73	<3–1600	LLE, derivatization, GC-MS	Berg et al. [9] (2000)
1997	The USA	1	215–230	Anion-exchange SR Empore disk, derivatization, HS-GC-ECD	Wujcik et al. [33] (1998)
1999	Canada	7	<0.1–170	Rotary evaporation, derivatization, LLE, GC-MS	Scott et al. [16] (2000)
1999	Malawi	1	4–15	Rotary evaporation, derivatization, LLE, GC-MS	Scott et al. [18] (2005)
1999	Canada	3	<0.5–350	Rotary evaporation, derivatization, LLE, GC-MS	Scott et al. [18] (2005)
1999	Chile	2	5–87	Rotary evaporation, derivatization, LLE, GC-MS	Scott et al. [18] (2005)
2001–2002	China	12	25–240	Rotary evaporation, derivatization, HS-GC-ECD	Zhang et al. [27] (2005)
1998–2004	The USA and Canada	206	3–2400	Rotary evaporation, derivatization, LLE, GC-MS	Scott et al. [17] (2006)
2007	Japan	4	39–76	WAX-SPE, ion-exchange HPLC-MS/MS	Taniyasu et al. [20] (2008)
2007–2008	China	32	46–970	Rotary evaporation, derivatization, LLE, GC-MS	Wang et al. [21] (2014)
2012	China	2	280 ± 68	Rotary evaporation, derivatization, LLE, GC-MS	Zhai et al. [25] (2015)
PFPrA					
1998–2004	The USA and Canada	206	< 0.1–120	Rotary evaporation, derivatization, LLE, GC-MS	Scott et al. [17] (2006)
2001	Canada	3	5.1–21	Rotary evaporation, derivatization, LLE, GC-MS	Scott et al. [32] (2006)
2006–2007	The USA	12	1.1–20	WAX-SPE, ion-exchange HPLC-MS/MS	Kwok et al. [39] (2010)
2007	China	5	1.1–3.1	WAX-SPE, Ion-exchange HPLC-MS/MS	Kwok et al. [39] (2010)
2007	Japan	4	8.9–10	WAX-SPE, ion-exchange HPLC-MS/MS	Taniyasu et al. [20] (2008)
2006–2008	Japan	31	0.9–45	WAX-SPE, Ion-exchange HPLC-MS/MS	Kwok et al. [39] (2010)
2008	India	2	0.2–0.3	WAX-SPE, ion-exchange HPLC-MS/MS	Kwok et al. [39] (2010)
2008	France	2	0.9–1.0	WAX-SPE, ion-exchange HPLC-MS/MS	Kwok et al. [39] (2010)

*LLE*, liquid-liquid extraction; *HS*, headspace

**Table 4:** Reported concentrations of TFA (ng/L) in surface water using different analytical techniques

Sampling year	Country	Number of samples (*n*)	Concentration range (ng/L)	Analytical technique	Reference (publication year)
1995–1996	Germany	47	10–630	Derivatization, LLE, GC-MS	Jordan and Frank [12] (1999)
1995	Austria	3	55	Derivatization, LLE, GC-MS	Jordan and Frank [12] (1999)
1995	Israel	9	200–2400	Derivatization, LLE, GC-MS	Jordan and Frank [12] (1999)
1996	Russia	3	35	Derivatization, LLE, GC-MS	Jordan and Frank [12] (1999)
1996	Brazil	3	<15	Derivatization, LLE, GC-MS	Jordan and Frank [12] (1999)
1996	Finland	4	210	Derivatization, LLE, GC-MS	Jordan and Frank [12] (1999)
1996	South Africa	21	<15–500	Derivatization, LLE, GC-MS	Jordan and Frank [12] (1999)
1995	Ireland	10	<10–70	Derivatization, LLE, GC-MS	Jordan and Frank [12] (1999)
1995	France	3	250	Derivatization, LLE, GC-MS	Jordan and Frank [12] (1999)
1996	Australia	3	200	Derivatization, LLE, GC-MS	Jordan and Frank [12] (1999)
1996–1997	The USA	66	13–470	Anion-exchange SR Empore disk, derivatization, HS-GC-ECD^a^	Wujcik et al. [24] (1999)
1997	Canada	14	<0.5–360	Rotary evaporation, derivatization, LLE, GC-MS	Scott et al. [16] (2000)
1996–1997	Switzerland	102	12–360	LLE, derivatization, GC-MS	Berg et al. [9] (2000)
1997	The USA	3	51–86	Anion-exchange SR Empore disk, derivatization, HS-GC-ECD^a^	Wujcik et al. [33] (1998)
1998–2000	Africa	5	1–5	Rotary evaporation, derivatization, LLE, GC-MS	Scott et al. [19] (2002)
1998–2000	The USA and Canada	8	51–99	Rotary evaporation, derivatization, LLE, GC-MS	Scott et al. [19] (2002)
2001	China	17	6.8–220	Rotary evaporation, derivatization, HS-GC-ECD	Zhang et al. [27] (2005)
2012	China	5	350–830	Rotary evaporation, derivatization, LLE, GC-MS	Zhai et al. [25] (2015)
2016	Germany	25	5400–140,000	Direct injection ion-exchange LC-MS/MS	Scheruer et al. [14] (2017)
–	Germany	43	Up to 17,000	WAX-SPE, mixed-mode ion-exchange HPLC-MS/MS	Janda et al. [11] (2018)
2017–2018	Sweden	8	<34–2700	Direct injection SFC-MS/MS	Björnsdotter et al. [35] (2019)

^a^Samples with conductivity > 500 μS were cleaned up by LLE prior to extraction

In surface water, the concentration of TFA ranged from < 0.5 ng/L [16] to 140 μg/L [14]. The highest concentration (140 μg/L) was observed in a river downstream a chemical industry producing fluorinated chemicals [14]. Higher concentrations of TFA has been observed in surface waters in industrialized areas compared with less industrialized areas [12]. Concentrations of TFA have also been reported in groundwater (< 5 ng/L to 7.5 μg/L) [9, 11, 12], ocean water (1–250 ng/L) [10, 12, 15, 22], fog water (20 ng/L to 2.2 μg/L) [13, 24, 33], in municipal (90–600 ng/L) and industrial (< 100 ng/L to 206 μg/L) wastewater effluent [9], in air (10 ng/L to 6.3 μg/L) [12, 23, 26], and in drinking water (16 ng/L to 11 μg/L) [9, 11, 16, 27]. Moreover, TFA has been reported in soil (< 0.1–9.4 ng/L) and conifer needles (< 2–420 ng/L) [18].

PFPrA has been reported in precipitation at concentrations ranging from < 0.1 to 120 ng/L [17, 20, 39] and in municipal wastewater influent (1.1–41 ng/L) and effluent (0.9–38 ng/L) [40]. TFMS has been recently reported in surface water and groundwater at concentrations up to 1 μg/L [41]. PFEtS has been reported in wastewater influent (1.4–17 ng/L) and effluent (0.08–11 ng/L) [40], and in drinking water at concentrations up to 0.9 ng/L [42]. PFPrS has been reported in wastewater influent (0.05–7.5 ng/L) and effluent (0.05–4.1 ng/L) [40], and in drinking water [42]. Furthermore, PFEtS and PFPrS have been reported in groundwater at military training sites at concentrations ranging from 11 ng/L to 75 μg/L and from 19 ng/L to 63 μg/L, respectively [43].

There is limited data presenting environmental concentrations of PFPrA, TFMS, PFEtS, and PFPrS. The potential sources and the environmental fate of these substances are therefore not yet well understood. However, with respect to their high polarity and high persistency, high concentrations in the aqueous environment could be expected. More research is required in order to gain knowledge about current environmental concentrations of these substances.

### Sources

A variety of sources of TFA together with a diversity of potential precursors and degradation pathways resulting in short- and long-range transport have been shown. Elucidating sources of ultra-short-chain PFAAs is important in order to sample the relevant matrices and prevent contamination in sampling and analytical determination. The relevance of the different sources may depend on location and be affected by factors such as urbanization, industries, hours of sunlight per day, etc. More research is required to determine the relevance of different sources as well as to identify yet unknown sources of TFA. Information about relevant sources of ultra-short-chain PFAAs other than TFA, i.e., PFPrA, TFMS, PFEtS, and PFPrS, is scarce. PFPrA likely originates from similar sources as TFA. High concentrations can be expected in the aquatic environment as a result of the structural properties as described in the previous section. With limited (TFA) or no (PFPrA, TFMS, PFEtS, and PFPrS) available information about human or aquatic toxicity, precatory measures should be taken and more studies are required for the identification of potential sources to the environment.

#### Trifluoroacetic acid

Atmospheric degradation of HFCs and HCFCs result in formation of TFA [44]. Measurements of TFA in the environment, mainly in precipitation and surface waters, have revealed that the observed environmental concentrations cannot be explained by the degradation of HFCs and HCFCs alone [12]. Furthermore, HFCs used in other applications besides cooling agents may also play a role in the environmental concentrations of TFA, as TFA is a degradation product of 2H-heptafluoropropane (HFC-227ea) used in fire extinguishers [45]. Formation of TFA can also occur via oxidation of precursor compounds, such as n:2 fluorotelomer alcohols (FTOHs) [46], that are major raw materials used in surfactant and surface protecting products; perfluoroalkane sulfonamide derivatives having four perfluorinated carbon atoms (e.g., perfluorobutane sulfonamide, FBSA) [2]; and *N*-methyl perfluorobutane sulfonamidoethanol (*N*-MeFBSE) [47], which is used as stain protectants for carpets, fabrics, and paper products. Atmospheric concentrations of TFA has been shown to be higher in spring and summer compared with autumn and winter [23, 48]. Wu et al. [23] showed that the airborne TFA measured in the north of China peaked in the afternoon and reached a minimum during early morning, following a diurnal cycle. These findings suggest that degradation of volatile precursors is a major source of airborne TFA.

TFA may also be formed during thermolysis of fluoropolymers in industrial and consumer products [49]. The direct formation of TFA during combustion would result in short-range transport resulting in locally elevated TFA concentrations in urban areas, whereas the indirect formation would occur via formation of intermediate propenes such as hexafluoropropene (HFP), which reacts with OH radicals in the atmosphere resulting in formation of TFA [49]. The second alternative would be a transport pathway of TFA to remote locations due to long-range transport of HFP before the formation of TFA as the atmospheric lifetime of HFP is 9 days [50]. Other potential sources of TFA include industrial wastewaters [9], hazardous waste management facilities, landfills, and firefighting training sites [35]. TFA has also been shown to be formed during ozonation of wastewaters that contain precursors such as plant-protecting agents and pharmaceuticals containing trifluoromethyl moieties [14]. There have been some studies investigating whether or not TFA is naturally occurring [10, 12, 15, 22, 37]. However, the topic is still under debate as existing results are contradictory and more research is required for a better understanding.

#### Perfluoropropanoic acid

Sources of ultra-short-chain PFAAs other than TFA are not as well studied and data is therefore limited. Sources and fate similar to TFA may be expected for PFPrA due to the structural similarities of these two compounds with PFPrA containing an additional CF_2_ compartment between the trifluoromethyl moiety and the carboxylic group. Similarly as for TFA, PFPrA is formed during atmospheric degradation of HFCs and HCFCs [51], via thermolysis of fluoropolymers [49] and by indirect formation by oxidation of precursor compounds such as n:2 FTOHs and FBSA derivatives via chain unzipping of intermediates [2]. Moreover, PFPrA has been shown to be a degradation product of perfluoro-2-methyl-3-pentanone (PFMP), a commonly used firefighting fluid [52]. Other sources include hazardous waste management facilities, landfills, and firefighting training sites [35].

#### Trifluoromethane sulfonic acid

TFMS, also known as triflic acid (TfOH), is a super acid widely used in organic synthesis [53]. The salt of TFMS with lithium is commonly used in lithium ion batteries [54]. There is limited research about potential sources of TFMS to the environment. Recently, TFMS was reported in water collected in connection to firefighting training sites, landfills and a hazardous waste management facility [35].

#### Perfluoroethane sulfonic acid

PFEtS was recently reported in aqueous film-forming foams (AFFFs) at concentrations ranging from 7 to 13 mg/L [43]. The same study also reported concentrations ranging from 11 ng/L to 7.5 μg/L in groundwater collected at military training sites. Furthermore, PFEtS was observed at high concentration (1.7 μg/L) in water collected in connection to firefighting training sites with known usage of AFFFs [35]. The findings by Barzen-Hanson and Field [43] suggest that PFEtS is present as a residual formulation and/or byproduct from products manufactured by the electrochemical fluorination method.

#### Perfluoropropane sulfonic acid

Similarly as PFEtS, PFPrS has been reported in AFFFs as well as in groundwater from military training sites. The concentration of PFPrS ranged from 120 to 270 mg/L in five AFFFs and from 19 ng/L to 63 μg/L in groundwater [43]. Moreover, PFPrS was observed at high concentration in water collected in connection to firefighting training sites with known usage of AFFFs [35]. The discovery by Barzen-Hanson and Field [43] suggest that PFPrS, just like PFEtS discussed above, is present as a residual formulation and/or byproduct from products manufactured by the electrochemical fluorination method.

### Human health and environmental concerns

The environmental fate of TFA has been studied for a few decades but there are still knowledge gaps that needs to be filled. It is well known that TFA enters the biosphere via wet deposition and that high concentrations can be expected in aqueous environments. Limited knowledge about sources and fate of the other ultra-short-chain PFAAs makes it complicated to estimate potential hazards of these. However, due to the structural similarities of these substances, similar environmental fate as for TFA might be assumed upon entering the biosphere. However, the global distribution may be different for the other ultra-short-chain PFAAs compared with TFA and PFPrA since there is no data on potential volatile precursors of TFMS, PFEtS and PFPrS. A few studies have investigated the accumulation in terminal aquatic systems [9, 24, 36] and the uptake and potential accumulation of TFA in plants [36, 55–57]. Uptake and accumulation in plants, of both TFA and the other ultra-short-chain PFAAs, might be hazardous for the environment and human health and should be further investigated in the near future as the environmental concentrations of ultra-short-chain PFAAs can be expected to increase with continued use of PFASs.

The toxicity of TFA to humans, animals, fish and algae has been evaluated and the lowest no observed effect level (NOEL) reported was 120 μg/L for a sensitive strain of algae [58]. Based on this data, it was concluded in 1999 that the current and estimated future environmental concentrations of TFA resulting from the degradation of HFCs and HCFCs (maximum 100 ng/L in precipitation in 2020) do not pose a threat to humans or the environment [58]. However, concentrations at least ten times higher than 100 ng/L have been observed in precipitation [9, 17, 22]. Berends et al. [59] evaluated the impact of TFA on the aquatic environment and concluded that a TFA concentration of 100 μg/L is safe for the environment. However, even if TFA has been commonly reported in surface waters and oceans at concentrations below 500 ng/L, a recent study has reported TFA in a river at a concentration higher than 100 μg/L [14].

The environmental concentrations of TFA are expected to increase in the future as a result of the Kigali amendment to the Montreal Protocol aiming in limiting the future use of HFCs. As a result of this agreement, HFC-134a which is the commonly used cooling agent in vehicles worldwide and a relevant source to TFA in the environment, will be phased out. A proposed replacement is HFO-1234yf (2,3,3,3-tetrafluoropropene), which has a shorter lifetime and higher conversion rate into TFA compared with HFC-134a. A few studies have estimated that concentrations of TFA in air and wet deposition will increase in the future assuming a complete shift to HFO-1234yf [60–62]. Results indicate that the TFA concentration in air will increase with at least a factor of 10 in some European areas [60]. The estimated TFA concentrations in wet deposition is lower than what has been considered a safe concentration for the most sensitive aquatic organisms [58]. However, TFA concentrations might reach hazardous concentrations in aquatic ecosystems and in plants as a result of accumulation [62].

Due to the high mobility and polarity of ultra-short-chain PFAAs, accumulation in water bodies can be expected. Point-source releases may result in elevated concentrations in drinking water. Recently, TFA has been observed in drinking water at concentrations up to 11 μg/L [11]. The currently used methods to purify drinking water does not remove PFASs, and even if activated carbon is used, short-chain PFASs are not effectively retained [63] and ultra-short-chain PFAAs are likely not retained at all [14, 35]. Neither ozonation nor chlorination resulted in reduction of TFA [14]. In the same study, a rapid break-through of TFA was observed with granular activated carbon (GAC) filters. Reverse osmosis removed TFA efficiently and might therefore be a useful technique for removing TFA and other ultra-short-chain PFAAs from water [14, 41].

There is limited data on TFA toxicity published and little is known about the toxicity of ultra-short-chain PFAAs other than TFA; more research is needed in order to assess the potential hazards for humans and for the environment. These substances are highly polar and do not have a potential for bioaccumulation. However, the persistence will result in continuously increasing concentrations in the environment, essentially in the aquatic environment and in drinking water. In the end, humans will be continuously exposed to elevated concentrations increasing the internal exposure. Therefore, due to the persistence of TFA and other ultra-short-chain PFAAs, along with expected increased environmental concentrations, continued attention is necessary. Furthermore, there has been an increasing interest on methods for TOP assay and extractable organic fluorine with the inclusion of ultra-short-chain PFAAs, since they can be formed as oxidation products and will contribute to the fluorine mass balance [29, 30]. Efforts should be made to increase the performance of the existing methods, in terms of extraction recovery, repeatability, and reproducibility, so that produced data can be comparable. Potential blank contamination sources should be evaluated and controlled.
